# Exploring Massive, Genome Scale Datasets with the GenometriCorr Package

**DOI:** 10.1371/journal.pcbi.1002529

**Published:** 2012-05-31

**Authors:** Alexander Favorov, Loris Mularoni, Leslie M. Cope, Yulia Medvedeva, Andrey A. Mironov, Vsevolod J. Makeev, Sarah J. Wheelan

**Affiliations:** 1 Department of Oncology, Division of Biostatistics and Bioinformatics, Johns Hopkins University School of Medicine, Baltimore, Maryland, United States of America; 2 Vavilov Institute of General Genetics, Russian Academy of Sciences, Moscow, Russia; 3 Research Institute of Genetics and Selection of Industrial Microorganisms, Moscow, Russia; 4 Department of Bioengineering and Bioinformatics, Moscow State University, Moscow, Russia; 5 Institute for Information Transmission Problems, Russian Academy of Sciences, Moscow, Russia; National Evolutionary Synthesis Center, United States of America

## Abstract

We have created a statistically grounded tool for determining the correlation of genomewide data with other datasets or known biological features, intended to guide biological exploration of high-dimensional datasets, rather than providing immediate answers. The software enables several biologically motivated approaches to these data and here we describe the rationale and implementation for each approach. Our models and statistics are implemented in an R package that efficiently calculates the spatial correlation between two sets of genomic intervals (data and/or annotated features), for use as a metric of functional interaction. The software handles any type of pointwise or interval data and instead of running analyses with predefined metrics, it computes the significance and direction of several types of spatial association; this is intended to suggest potentially relevant relationships between the datasets.

Availability and implementation: The package, GenometriCorr, can be freely downloaded at http://genometricorr.sourceforge.net/. Installation guidelines and examples are available from the sourceforge repository. The package is pending submission to Bioconductor.

This is a *PLoS Computational Biology *Software Article

## Introduction

Manual exploration of high-dimensional whole-genome datasets is possible, to a limited extent, with newer, high-capacity genome browsers. While biologists can browse their data and can often suggest relevant hypotheses for statistical testing, fully informed and thorough data exploration is impossible to do by eye.

A common theme in biological experiments is that the nucleotide-level proximity of a set of genomic regions (points or intervals) to genome annotations or to other experimentally derived data sets (such as coverage peaks, mutation locations, and breakpoints) is a useful proxy for a functionally relevant or otherwise interesting interaction. For example, the well established overlap of CpG islands with the promoter regions of genes [1] is critically related to the gene-silencing mechanism of DNA hypermethylation.

While using spatial proximity to infer functional relationships is a valid approach in many cases, this is not necessary for functional interaction, as chromatin is flexible and many activating and repressive marks act at a distance [2], so ideally any software that attempts to automatically uncover important relationships should be sensitive to these interactions as well. Others have given thought to examining some of the interactions that we will discuss, (for a review see Bickel et al, 2009 [3]); however, the only software for performing such analyses focuses on overlapping features [4].

Here we present a method for identifying whether two sets of intervals are spatially correlated across a genome, detected as a deviation from a nonuniform distribution of one set of intervals with respect to the other. This is not a trivial task, computationally or conceptually. Many different spatial rearrangements are possible, each with different biological implications and each requiring specialized statistical analyses. The software performs all analyses on each input, so that a variety of biologically significant relationships are queried. This includes looking for proximity, looking for uniform spacing, looking for increased or decreased overlaps of intervals or points, and presenting the data in a way that a biologist can understand. Results from each test are provided for each chromosome from the dataset and for the entirety of the dataset, which in most cases is the entire genome. As we have found that asking “is A related to B” is completely different from asking “is B related to A,” we encourage users to not only perform all comparisons but to perform them in both orientations.

## Design and Implementation

### Overview and general considerations

GenometriCorr is written in R (using S4 classes) and makes use of the Bioconductor [5] packages IRanges and GRanges to create sets of intervals that are then compared. The input data can be in a variety of commonly used biological data formats, and the core functions work from a configuration file that sets parameters in a straightforward, easy to edit format that can be archived to ensure reproducibility. We provide a Tk interface so that non-programmers may access the functions via straightforward menus, and we also provide a Galaxy [6] plugin that runs the analysis in an environment widely used by biologists, in which the results may be explored more thoroughly. Finally, we provide two auxiliary methods for output, so that graphical results can be obtained in addition to the statistics produced by the main function. The configuration and the result, GenometriCorrConfig and GenometriCorrResult are designed such that once a configuration file has been read, the software proceeds with a simple run.config call.

The main function, GenometriCorrelation, implements various statistical approaches to assess whether the positions of two sets of intervals are associated in genomic space. As stated above, almost all of the tests are asymmetrical, in that one of the two interval sets is considered to be a reference, fixed in the genome, while the other set, the query, is evaluated statistically with respect to the fixed reference. The results can be very different if the reference and query sets are swapped, as shown below. In essence, each of the tests is designed to evaluate whether the spatial distribution of the query intervals is independent of the positions of the reference intervals, and each test is sensitive to a different aspect of known biological relationships.

**Figure 1:**
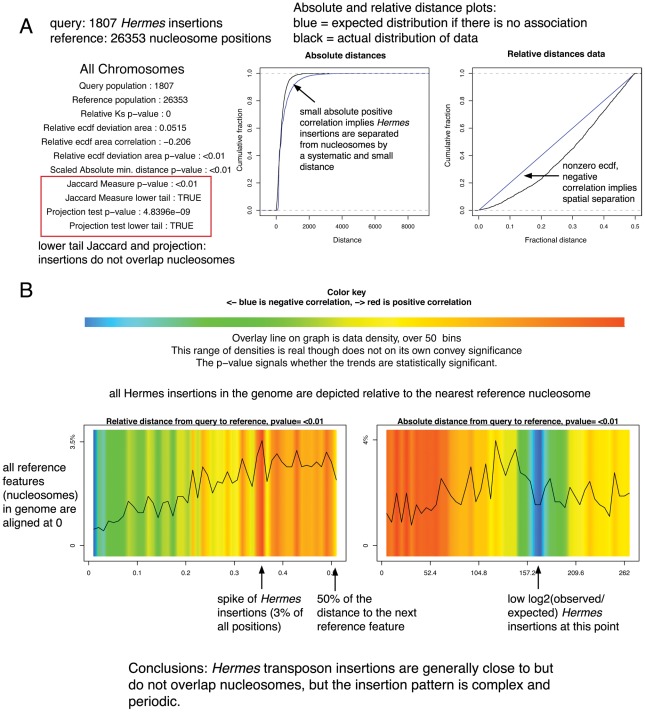
Two types of graphic output are available. (A) A statistical summary and ECDF plots. (B) A graphical interpretation of the spatial relationships. The query features are depicted along the plot according to their distance to a reference feature; the colors indicate deviation from the expected distribution while the overlay line indicates the density of the data at each absolute or relative distance. The data density mirrors but is independent from the log-odds colors; at small distances in the absolute distance plot the data density is higher than expected but this represents a very small percentage of the total query points.

Two types of graphical output are produced. Calling the graphics.plot() function produces a straightforward statistical summary and ECDF plot for the relative and absolute distances for each chromosome and/or the entire genome. Summary results for all chromosomes together are displayed in a window and results for the individual chromosomes are written to a PDF. The visualize() function produces a more elaborate and biologist-friendly color-coded density plot, intended to represent areas of high and low absolute and relative distance correlation; again, summary results appear in a window and chromosome-by-chromosome results are written to a PDF. The two types of output are shown in Figure 1, along with detailed descriptions of the features of each. These data are Hermes transposon insertions in the yeast genome; this transposon generally inserts into nucleosome free regions [7].

An important consideration here is that two sets of genomic features may only be correlated in one direction. As an example, we found all NF-kappa-B (NFKB1) sites using a simple exact string search of the human genome and correlated their positions to the positions of all RefSeq gene [8] start sites. NF-kappa-B is a family of transcription factors critical in many processes, including immunity, inflammation, and cell growth [9].

**Figure 2:**
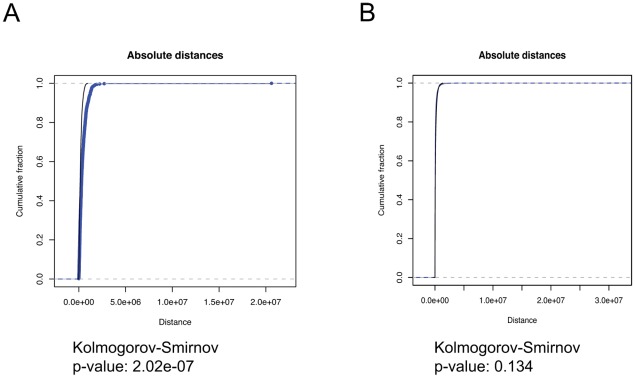
NFkappaB sites vs human RefSeq promoter start sites. Query and reference colors as in Figure 1. (A) NFkappaB as the query gives a significant Kolmogorov-Smirnov association and anticorrelation that is visible from the graph, in absolute distances. (B) Correlation in the reverse direction suggests no significant relationship between the two classes of sites.

As there are nearly five times as many transcription start sites as potential NF-kappa-B sites, most transcription start sites will not be near a NF-kappa-B site even if they are perfectly correlated, while the NF-kappa-B sites will nearly all be close to transcription start sites. Figure 2a depicts the excellent correlation between human NF-kappa-B sites to transcription start sites (same distribution, perfect absolute distance correlation), and Figure 2b depicts the poor (and not statistically significant) correlation of transcription start sites to NF-kappa-B sites (absolute distance indicates a separation, K-S not significant). As this level of asymmetry is common, if not expected, in biological datasets, we recommend performing all comparisons in both directions, using each dataset as the fixed set in turn. While the relevant comparison is not known a priori, the results of the two comparisons will clearly indicate whether the relationship is asymmetric.

Many of the tests we used are performed on pointwise representations of the intervals rather than on the intervals themselves. When the input includes intervals, the midpoints of these intervals are used for those analyses. Very large intervals may relate to genomic features in different ways, depending on whether we examine their start points, stop points, both boundaries, or just a point in the middle. Rather than trying to address this ambiguity or to randomly guess at what the user hopes to do, we expect the user to specify the points when the exact point is important, and we use the midpoint when the user inputs an interval.

**Figure 3:**
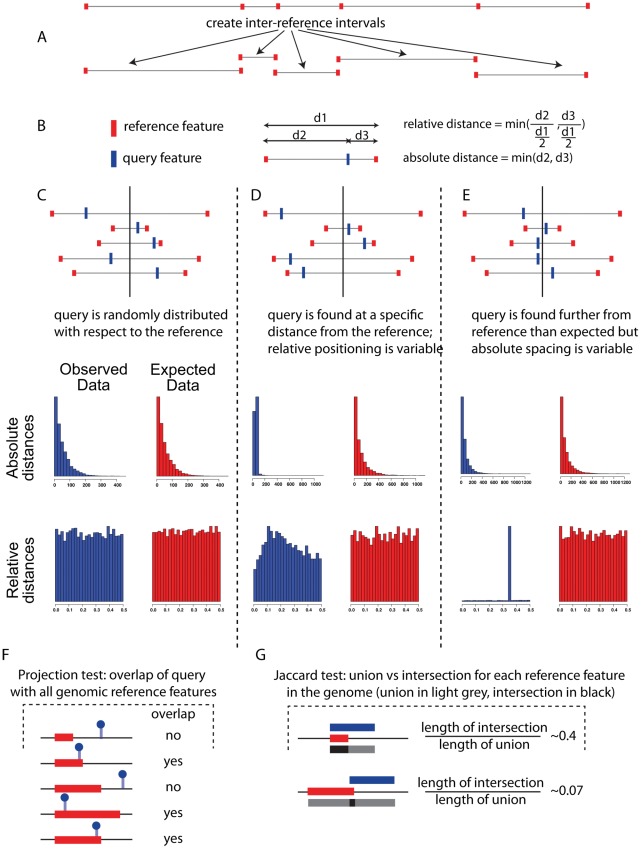
A schematic of the various tests implemented in the software package, showing when certain tests are most useful. (A) depicts the intervals created in silico and (B) shows how the query distances are evaluated within the intervals. (C) depicts a random distribution of query versus reference intervals; here the observed and expected distances for both the absolute and relative tests are the same. In (D) we show a relationship best uncovered by the absolute distance test; useful especially for small genomes, this test determines whether the query and reference are often separated by a fixed distance. In (E), the query points are consistently far away from the reference points, so the relative distance test will be significant, while the absolute distances are not significant in this case. Interestingly, the query intervals are variable enough in size that even though the query and reference points are usually separated, the absolute distances between them vary widely in size, including some fairly small distances. (F) demonstrates the projection test, which evaluates whether pointwise data falls consistently inside or outside of a set of intervals. Finally, in (G) we see the Jaccard test, which looks for significant overlaps between datasets by evaluating the ratio of the intersection of the datasets (dark grey) to the union of the datasets (light grey). Perfect correlation will give a ratio of 1, and perfect anticorrelation will result in a ratio of zero.

We have developed and tested four useful and relevant metrics, which will be discussed below: the relative distance test, the absolute distance test, the Jaccard test, and the projection test, intended to measure a variety of biologically relevant correlations. In Figure 3 we summarize the metrics and their uses, and in subsequent figures we demonstrate the utility of each type of test, using both published and novel observations. Each figure shows both a standard histogram representation of the relationships between the query and reference sets, in addition to the results and p-values generated by our software. As a strong correlation between the query and reference may involve just a subset of a very large number of points, a histogram of the absolute or relative distances can be uninformative, while the tests performed by the software are sensitive to true correlations within large and overall not strongly correlated datasets. All p-values cited are computed by the GenometriCorr functions. For each test we describe a published dataset for which the test is particularly useful. Table 1 and table 2 summarize the results.

**Table 1:** Summary of all correlations performed in these experiments.

	Direction	Relative Kolmogorov-Smirnov p–value	Relative ECDF area correlation	Relative ECDF deviation area p–value		
					C	F
Human transcription start sites (T) versus NF-kappa-B sites (N)	T→N	2e−07	0.015	<0.001	√	
	N→T	0.13	0.012	0.072	√	
L1 elements(T) versus Splice Sites (S)	T→S	∼0*	−0.03	<0.001		√
	S→T	∼0	−0.16	<0.001		√
Promoter sites (P) versus Promoter plus spikein (S)	P→S	∼0	0.25	<0.001	√	
	S→P	∼0	0.25	<0.001	√	
H3K4me3 histones (H) versus Promoters of actively transcribed genes (P)	H→P	∼0	0.22	<0.001	√	
	P→H	∼0	0.02	<0.001	√	
CpG Islands (I) versus Coding sequences (C)	I→C	∼0	−0.195	<0.001		√
	C→I	∼0	−0.012	<0.001		√

*: ∼0 means that the default R precision for KS test p-value is not enough to distinguish the value from 0.

**Table 2:** Continued summary of correlations performed in the experiments described.

	*Direction*	Scaled Absolute minimal distance *p–value*			Jaccard Measure *p–value*			Projection test *p-value*		
			C	F		O	N		O	N
Human transcription start sites (T) versus NF-kappa-B sites (N)	T→N	<0.001	√		<0.001		√	0.0091		√
	N→T	<0.001	√		<0.001		√	∼0*		√
L1 elements(T) versus Splice Sites (S)	T→S	<0.001		√	<0.001		√	∼0		√
	S→T	<0.001		√	<0.001		√	∼0		√
Promoter sites (P) versus Promoter plus spikein (S)	P→S	<0.001	√		<0.001		√	∼0		√
	S→P	<0.001	√		<0.001		√	∼0		√
H3K4me3 histones (H) versus Promoters of actively transcribed genes (P)	H→P	<0.001	√		<0.001	√		∼0	√	
	P→H	<0.001	√		<0.001	√		∼0	√	
CpG Islands (I) versus Coding sequences (C)	I→C	<0.001		√	<0.001		√	∼0		√
	C→I	<0.001		√	<0.001		√	∼0		√

*: ∼0 means that the default R precision for the binomial test is not enough to distinguish the value from 0.

P-values are shown for all tests in both directions (using one dataset as the query and the other as the reference, then reversing). For each test, we have indicated whether the relationship between the datasets is positive or negative; for the relative and absolute distance tests this is written as “close (C)” vs “far (F)” and for the Jaccard and projection tests it is written as “overlapping (O)” vs “nonoverlapping (N).”

### Relative distance test

**Figure 4:**
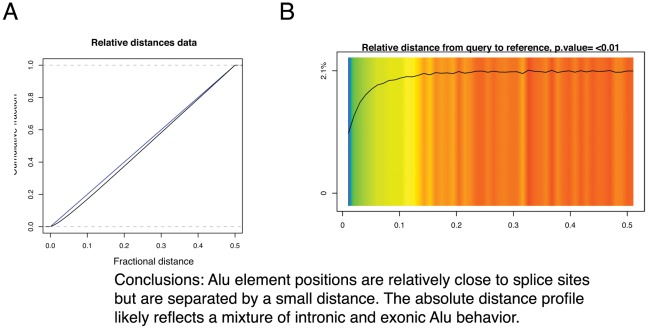
Alu elements vs splice sites in the graphics.plot() output (A) and in the visualize() output (B). Alu elements are consistently located at a variable but always nonzero distance from splice sites. Query and reference colors as in Figure 1.

The relative distance test measures whether two sets of positions are closer together or further apart than expected, where the exact distances are not as important as the relative relationship. For example, a recent publication [10] reported that transposable elements found in genes tend not to be located near splice sites. We tested this association with the GenometriCorr software and found that, first, the two entities do not overlap (the Jaccard and projection tests, summarized in figure 3 and described in detail later, are both significant and in the lower tail) and that both the relative and absolute distance tests show a correlation. Upon closer examination, the transposable element and splice site positions are actually negatively correlated in terms of relative distance; that is, the two types of genomic features tend not to co-occur but are consistently spaced apart, though not by a uniform distance (Figure 4). Results are shown for Alu elements but the relationship holds true for both L1 and Alu elements, in agreement with the reported trends.

The idea behind the relative distance test is that if the query locations are independent of the references, then the relative position of each query point, with respect to the adjacent reference points, will have a uniform distribution. Thus, the null distribution for relative distance test as formulated here is simply a straight line at y = 0.5. For this test, intervals are represented as points, located at the midpoint of the interval, so that the size of intervals and overlap between query and reference are not included in the analysis.

For each query point, 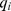, we identify the flanking reference points, 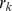 and 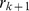, and calculate the relative distance 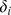 = (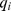,−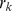)/(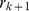,−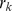), comparing this to a uniform null distribution. More formally, the “relative distance” 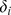 for the *i*-th query point is:
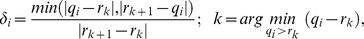
and under the null, the 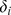
's would be distributed uniformly in [0, 1/2].

Two different tests are available to evaluate the uniformity of the distribution the 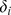
's. The first and simplest is the standard Kolmogorov-Smirnov test, assessing the maximum difference between CDFs.

A permutation-based test is carried out as well, to more comprehensively compare the two cumulative distribution functions using the area of the region in which they differ as the test statistic. Here 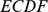 (*Empirical Distribution Cumulative Function*) designates the observed distribution of relative distances 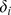, while 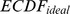 describes the expected distribution under the uniform, null distribution, which is a straight line. The area between the 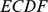 and 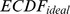 is then calculated as:
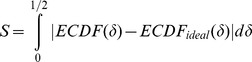
and a *p-value* for S obtained by permutation, in which we randomly draw 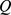 (number of query points) values from the ideal uniform distribution of 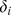 and calculate the area 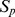.

The integral of the difference between the *ECDF* and the *ECDF_ideal_* also permits us to derive a natural measure of association for the two sets of intervals. The sign of the integral indicates the direction of the correlation, so the positive sign indicates that 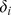
's tend to be low and thus the query intervals are attracted to reference intervals while, vice versa, the negative sign suggests that query intervals avoid reference intervals. With appropriate rescaling, we define a correlation-like measure:
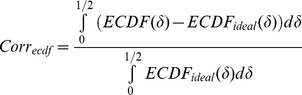
to express this. The 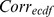 equals zero for independent query and reference; it equals 1 if each query point coincides with a query point and, finally, it equals 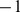 if each query point falls in the middle of the reference gap.

### Absolute distance test

**Figure 5:**
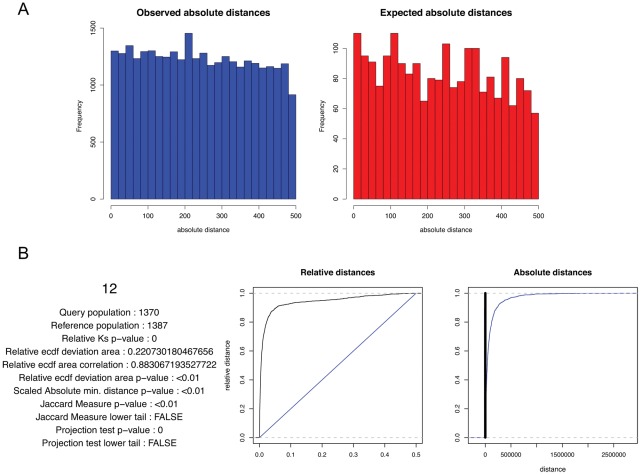
A toy example of absolute distance correlation. (A) Histograms of the observed and expected ranges of minimum distances between the reference and query. (B) GenometriCorr's simple plot for the same data. Query and reference colors as in Figure 1.

In some cases, particularly in small genomes in which reference points are closely spaced, the relative distance test produces misleading results. For example, if the promoters in a genome are generally found roughly 100–1500 bases apart (for example, yeast), an element that is positioned consistently 500 bp from promoters will look uncorrelated with promoters in relative terms, as it will sometimes be extremely close to a promoter that is not the one it is functionally related to, thereby diluting the distribution of query-reference distances with many incorrect data points. In these situations the absolute distance test is useful. We created a toy dataset for this analysis, first taking the positions of the start points of all human promoters (31083 sites), creating a new set of small intervals placed randomly from 10–10000 base pairs from each promoter start, and adding an additional 3000 small intervals randomly placed between 75 and 100 bp from a promoter. We then compared these intervals to the actual promoter intervals in the human genome, and the software uncovered the signal of the fixed distance points within the shifted points, whereas a simple histogram approach fails (Figure 5). The null distribution for the absolute distance test depends on the data. If the inter-reference intervals are somewhat randomly distributed, then the null distribution will be exponential, but if the inter-reference intervals are constrained somehow, the null distribution will have a very different shape.

As in the relative distance test, intervals are represented by their midpoints.

The statistic used is very simple and intuitive. We suppose there are 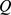 query points and 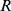 reference points on a chromosome, and for each query point 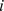, find the distance to the closest reference point, scaled by the expected inter-reference gap for the chromosome
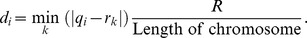
The final test statistic is the mean value of *d_i_*

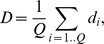
which characterizes the spatial association between the query and the reference points. The lower it is, the closer together they tend to be.

The *p-value* is obtained by permutation test. At each iteration, we draw 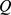 simulated query points uniformly distributed along the chromosome and calculate the permuted statistic 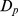. The *p-value* is the proportion of permuted statistics that exceed the observed 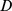. As implemented in the package, the test is two-sided and returns both the *p-value* and the direction of the association.

### Projection test

**Figure 6:**
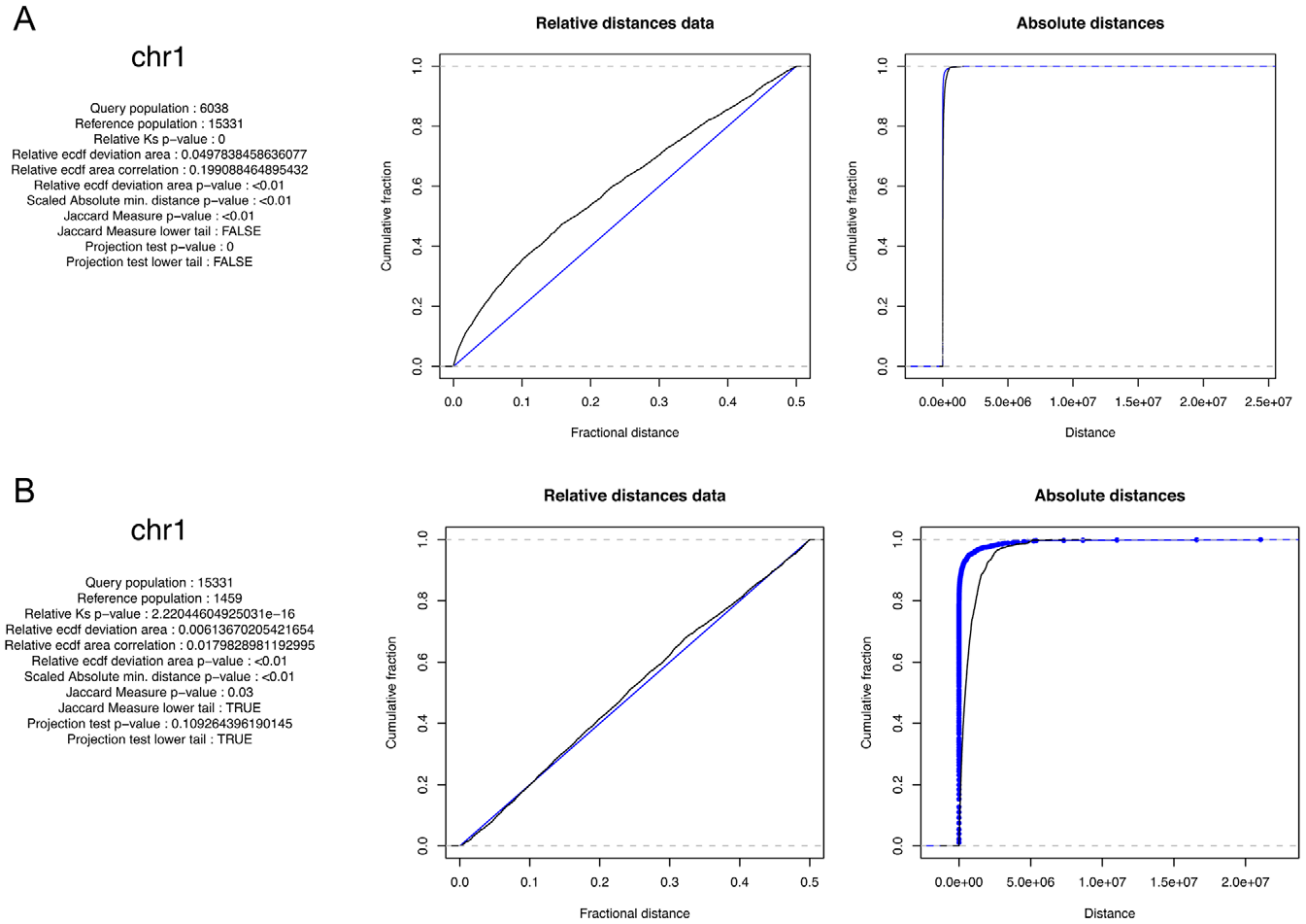
Promoter positions from highly expressed genes (as given from mRNAseq data) and histone ChIP data recently available from the Roadmap Epigenomics Project [**8**]. (A) H3K4me3 versus highly expressed genes. (B) H3K27me3 versus highly expressed genes. Query and reference colors as in Figure 1.

Another test included in the software is the projection test. As seen in figure 3, this tests whether pointwise data overlap interval data in a significant way. To confirm the biological relevance of this test we examined data from the Roadmap Epigenomics Project [11]. Using the RNAseq data and histones H3K27me3 and H3K4me3 ChIP data from UCSF-UBC (GEO accessions GSM484408 (RNAseq), GSM428295 (H3K27me3), and GSM410808/GSM432392, (replicates for H3K4me3)), we used the projection test to examine the relationship between the two histone marks and the promoters of the most highly expressed genes. The software was able to determine that the H3K4me3 marks significantly overlap the gene positions (Figure 6A) and the H3K27me3 marks are significantly underrepresented near active genes (Figure 6B). Note that in both cases the projection test is highly significant, but in opposite directions; for the H3K4me3 data the projection test is in the lower tail, indicating significant overlap, while the opposite is true for the H3K4me3 data, indicating very little overlap with promoters of active genes.

The projection test is the methodologically simpler of the two overlap tests in the package; the other, the Jaccard measure, is discussed below. For this test, query intervals are still represented as midpoints, but the reference should be a set of intervals. If the query points are independent of the reference, then the probability that any one query point is contained in a reference interval is the proportion of the chromosome covered by reference intervals:
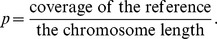
The total number of query points 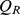 contained in reference intervals has a binomial distribution,
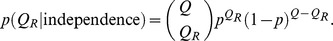
A standard two-sided binomial test is used to evaluate statistical significance. The test is unlikely to be informative if the genomic coverage of the reference intervals is very close to 0 or 1. ; here the p-values will be extremely high.

### Jaccard test

**Figure 7:**
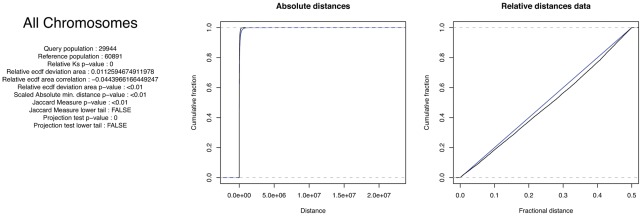
Human genomic CpG islands from Wu et al [**9**] correlated with the positions of coding sequences in the human genome. Query and reference colors as in Figure 1.

The Jaccard test measures overlaps between two interval sets by measuring the extent of intersection between two interval sets, divided by the length of their union. Thus, two datasets that overlap perfectly have a union that is equal to their intersection, and the ratio is 1. This proves to be a very useful measure for biological data, as demonstrated in Figure 7, in which CpG islands [12] are compared with coding sequences in the human genome. Comparing the CpG islands with the coding sequences we see that the two interval sets overlap much less than expected, given the amount of the genome that each occupies, and this anti-correlation is statistically significant. This is expected, as CpG islands generally occur in promoters and other non-genic regions.

The Jaccard statistic is calculated on intervals rather than points, and is the ratio of the number of nucleotides in the intersection of the reference and query, and the total number of nucleotides spanned by the reference and query together.

More formally:

The Jaccard statistic, 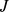, evaluates interval sets 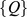 and 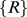 that are sets of chromosome positions that are covered by query and reference intervals, respectively.


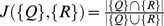
 where 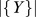 denotes the size of a set Y.

The *p-value* and the direction of difference from the null hypothesis (that the positions of 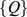 and 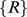 are independent) are obtained by permutation. Each permutation randomizes the query intervals uniformly across the chromosome, maintaining the spacing between intervals.

### Comparisons limited to genomic subsets

An investigator may want to explore correlations within defined intervals rather than genomewide; for example, when looking at binding sites within and very close to genes, the correlation between these sites will be extremely high genomewide because they are constrained to small intervals, but upon examination of the sites within genes, there may be no correlation at all. For this reason we provide methods that restrict the correlations to intervals defined by the investigator and that can be set up from within the configuration file.

## Results

**Figure 8:**
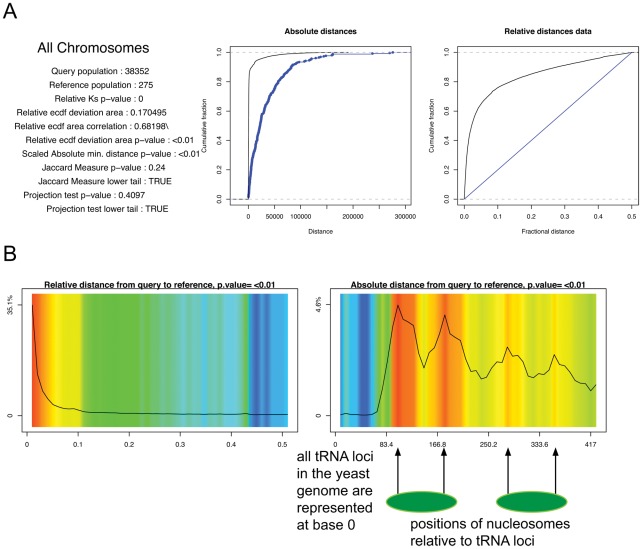
Ty1 retrotransposon insertion sites vs tRNA genes in the yeast genome. (A) ECDF plots (B) Graphic display. Arrows mark Ty1 insertion sites at nucleosome-occupied positions near tRNA genes. Nucleosomes are in green. The colored graph contains several regions of high observed/expected Ty1 insertions (red colors), and the black line indicates a high density of Ty1 insertions, as well, in these regions. Relative to the tRNA position, the Ty1 insertion sites are most dense inside the nucleosome occupied regions. Query and reference colors as in Figure 1.

We tested the software on our own high-dimensional data, a set of cloned insertion sites of an exogenously supplied Ty1 retrotransposon in the gene-rich yeast genome, for which we were trying to determine Ty1 targeting specificity. After sequencing and mapping it was clear that the insertions cluster near tRNA genes but do not generally insert into these genes, as seen in Figure 8A. Figure 8B displays the very complex relationship between Ty1 and tRNA promoters; the insertions occur at very specific points along nucleosome-bound DNA and follow a reproducible periodic pattern. On further examination we were able to map the insertion sites precisely to the nucleosome surface, as we have previously reported [13].

The examples provided here illustrate the range of biological questions that can be addressed with our software. A particularly compelling feature of the package is that negative correlations (overlap or proximity much less than expected if the query and reference were unrelated) are reported, meaning that correlations between factors that act at a distance are detectable. Also, we observe that absolute and relative distances are both important and may measure different phenomena; thus the software provides appropriate tests for these correlations as well.

We do not intend for our software to supplant the role of either the biologist or the statistician in a team of investigators working on high throughput sequencing or microarray data. Rather, by determining the statistical significance of genomewide interactions, the software serves as a hypothesis generator, enabling all investigators to begin validating associations that are likely to be real, much earlier than they would have otherwise.

**Figure 9:**
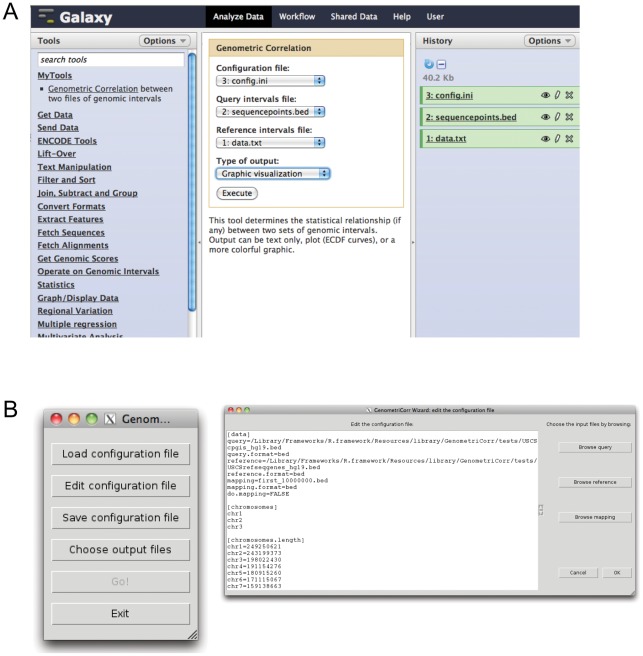
A) The Galaxy interface to GenometriCorr. B) The Tk interface to GenometriCorr. Instructions for using both are found on the website.

We do not provide a built-in method for retrieving query and/or reference features that may conform to a configuration suggested by the correlation methods. As we provide methods to use the software from within the Galaxy interface (below, and Figure 9), users with minimal computational experience can create any desired subsets using the many tools available in that environment. More computationally experienced users will have no trouble extracting query and reference intervals and overlapping these intervals as suggested by the correlation output.

GenometriCorr can be customized for use with any genome and any type of point or interval data.

## Availability and Future Directions

GenometriCorr is available, along with examples and installation guidelines, from http://genometricorr.sourceforge.net/. The software is written in R and can be used from the R command line, through a Tk graphical interface, or through the Galaxy interface; all of these options are documented on the site.

In future work we plan to implement the generalized Jaccard measure, which can handle sparsely distributed query and reference sets. Moreover, the generalized Jaccard measure can account for intervals that have a weight or other numerical value, in addition to coordinates. This weight can denote anything from multiplicity of coverage to experimental confidence.
